# ﻿Four new species of the genus *Pseudoeupolyphaga* Qiu & Che, 2024 (Blattodea, Corydioidea, Corydiinae) from Yunnan, China

**DOI:** 10.3897/zookeys.1261.168015

**Published:** 2025-12-02

**Authors:** Yi-Ming Ren, Wei Han, Yan-Li Che, Ji-Rui Wang

**Affiliations:** 1 Key Lab for Biology of Crop Pathogens and Insect Pests and Their Ecological Regulation of Zhejiang Province, College of Advanced Agricultural Sciences, Zhejiang Agriculture & Forestry University, Lin’an, Zhejiang 311300, China; 2 College of Plant Protection, Southwest University, Chongqing 400715, China; 3 Key Laboratory of Agricultural Biosafety and Green Production of Upper Yangtze River (Ministry of Education), Southwest University, Chongqing 400715, China

**Keywords:** Cockroach, COI, Dictyoptera, Polyphagini, species diversity, taxonomy

## Abstract

Four new species within the genus *Pseudoeupolyphaga* are described and illustrated: *Pseudoeupolyphaga duani* Ren & Han, **sp. nov.**, *Pseudoeupolyphaga menglianensis* Ren & Han, **sp. nov.**, *Pseudoeupolyphaga spelunca* Ren & Han, **sp. nov.**, and *Pseudoeupolyphaga vestis* Ren & Han, **sp. nov.** Morphological variants are recorded for the species *Pseudoeupolyphaga pilosa*. These findings further enhance the documented diversity and distribution patterns of *Pseudoeupolyphaga* in Southwest China.

## ﻿Introduction

*Pseudoeupolyphaga* Qiu & Che, 2024 (Corydioidea: Corydiinae), a genus endemic to China, currently represents the most species-rich group within this superfamily in the country (Han et al. 2024a, 2024b). Currently, 21 species and four subspecies are recognized (Han et al. 2024a). Species of this genus are predominantly concentrated in the southwestern regions of China, particularly in Yunnan and Sichuan provinces, and the Tibet Autonomous Region. Only the type species, *P. yunnanensis* (Chopard, 1929), exhibits sporadic occurrences in Guangxi, Guizhou, Jiangxi, Gansu, Qinghai, and Inner Mongolia (Qiu et al. 2018; Han et al. 2022, 2024a).

This genus is characterized by pronounced morphological convergence. Only a limited number of female adults exhibit a few diagnostic characteristics, rendering species identification largely reliant on morphological variation in males (Qiu et al. 2018; Han et al. 2024a). The tegmina of males are predominantly light yellow to yellowish brown in ground color, bearing variably sized and densely distributed brown to black markings—except in *P. fusca* (Chopard, 1929), which possesses uniformly black tegmina lacking markings. This forewing patterning constitutes the primary diagnostic character for species delimitation. Additionally, the male pronotum is typically transversely elliptical, ranging in color from yellowish brown to dark brown, with the width of its anterior whitish margin serving as a diagnostic character for certain species. The abdominal color pattern in males, characterized by a gradient or mottled transition from yellowish brown to dark brown, also aids identification. In contrast, the male genitalia structure—commonly utilized in traditional cockroach taxonomy—provides minimal taxonomic utility within this genus (Qiu et al. 2018). Similarly, female genitalia offer negligible diagnostic value (Han et al. 2024a, 2024b). Although mitochondrial COI (cytochrome *c* oxidase subunit I) gene sequence analysis helps resolve species identification for most species, ambiguous species boundaries persist in some specimens when integrating morphological and molecular data (Han et al. 2022, 2024a). This may indicate potential cryptic species diversity, or oversplitting of species; these two extreme situations might even coexist within the genus, underscoring an urgent need for refined species boundary delineation.

The main characteristics that distinguish this genus from other genera in Polyphagini are: (i) male body (excluding tegmina) usually less than 20 mm, tegmina with maculae; (ii) male right phallomere larger than the left phallomere; (iii) female apterous and robust; the middle of the posterior margin of the supra-anal plate is protruded; and (iv) female spermatheca has one large ampulla. The last point is the distinctive feature of *Pseudoeupolyphaga* species.

Yunnan Province is the region with the richest species diversity within this genus; it harbors records of thirteen species and two subspecies. The region’s complex mountain systems facilitate geographic isolation, promoting allopatric speciation. Utilizing recently collected specimens from Yunnan, this study integrates morphological and molecular phylogenetic evidence to describe four new species. This work substantially enhances our understanding of *Pseudoeupolyphaga* diversity in Yunnan and provides crucial material for elucidating mechanisms of species divergence and clarifying the taxonomic boundaries within the genus.

## ﻿Material and methods

All specimens examined in this study are deposited in the Insect Collection of Zhejiang Agricultural and Forestry University (ZAFU) and the College of Plant Protection, Southwest University (SWU). Terminology for external morphology follows Roth (2003); for male genitalia, Klass (1997).

The last three or four abdominal segments were excised, immersed in 10% KOH solution, and heated for 20 minutes to dissolve adipose tissue. Subsequent procedures, including dissection of males and their morphology, DNA extraction, PCR amplification, and sequencing, follow the protocols described by Han et al. (2022). A total of 45 COI sequences were analyzed, including 12 newly obtained sequences. Among these sequences, 43 sequences represent 21 species and subspecies within the genus *Pseudoeupolyphaga*, along with one unidentified species, while two sequences serve as outgroups. Additionally, four species and three subspecies from this genus were excluded from the analysis due to the absence of molecular data. All new sequences have been deposited in GenBank under accession numbers PX533468 to PX533480 (Table 1). The genetic distances and phylogenetic analyses followed the protocols described in Han et al (2024a). Specifically, all COI fragments were aligned using MEGA 11 (Kumar et al. 2016). Interspecific and intraspecific genetic distances were calculated under the Kimura 2-parameter (K2P) model (Kimura 1980). Maximum-likelihood (ML) analyses included 10 independent tree searches, each executed with 10,000 ultrafast bootstrap (UFBoot) replicates, after which we selected the highest likelihood result. The optimal partitioning scheme and nucleotide substitution models were determined using PartitionFinder v. 2.1.1 (Lanfear et al. 2017) under default parameters (COI_pos 1: SYM+I+G; COI_pos 2: TRN+I+G; COI_pos 3: GTR+G).

**Table 1.** Samples used in species delimitation.

Species	Abbreviation	GenBank ID	Collection information	Remark
*P. spelunca*	PseuSpel1	PX533470	Dashitou Village, Pu’er City, Yunnan; 19 Dec. 2024; Hang Qiu	Female
	PseuSpel2	PX533471	ibid	Male
	PseuSpel3	PX533478	ibid	Female
	PseuSpel4	PX533473	ibid	Nymph
	PseuSpel5	PX533472	ibid	Nymph
*P. vestis*	PseuVest1	PX533474	Liushun Township, Pu’er City, Yunnan; 24 Dec. 2024; Quan-Fu Duan	Female
	PseuVest2	PX533475	ibid	nymph
*P. menglianensis*	PseuMeng1	PX533468	Mengwai Village, Pu’er City, Yunnan; 6 Mar. 2025; Zhong-Hong Luo	nymph
	PseuMeng2	PX533469	ibid	nymph
*Pseudoeupolyphaga* sp1	PseuSp1	PX533480	Luding County, Ganzi Tibetan Autonomous, Sichuan; 27 Mar. 2025; Ding Zhou	Female
*P. duani*	PseuDuan	PX533476	Jinggu County, Pu’er City, Yunnan; 1 Jun. 25; Luo-Jiang Liu	nymph
*P. pilosa*	PseuPiloXT1	PX533477	Xiangtu Villaga, Jianchuan County, Yunnan, 25 Apr. 2025, He Zhang	Female
	PseuPiloXT2	PX533479	ibid	nymph
	PseuPiloLDT	PQ059681	Luodatang countryside, Yunnan; 25 Jul. 2022; Wei Han, Xin-Xing Luo, Lin Guo	female
	PseuPiloWBS	PQ059690	Wenbi Mountain, Yunnan; 24 Jul. 2022; Wei Han, Lin Guo	male
	PseuPiloYL	PQ059682	Lanyue Valley, Yunnan; 24 Jul. 2022; Wei Han, Lin Guo	male
	PseuPiloWX	OP215882	Pantiange Township, Weixi, Yunnan; 21 Aug. 2015; Lu Qiu	male
*P. fengi fengi*	PseuFengZXS	PQ059693	Zixi Mountain, Yunnan; 31 Jul. 2022; Wei Han, Xin-Xing Luo	female
	PseuFengDHS	PQ059679	Dahei Mountain, Sichuan; 22 Jul. 2022; Wei Han, Xin-Xing Luo	female
	PseuFeng1	OP215870	Mt. Zixishan, Chuxiong City, Yunnan; 7 Jul. 2012; Dong Wang	male
	PseuFeng2	OP215871	ibid	male
*P. simila*	PseuSimiMYL	OP215883	Miyaluo Town, Li County, Sichuan; 6 Oct. 2019; Lu Qiu, Hao Xu, Zhi-Teng Chen	female
	PseuSimiDGC	PQ059676	Dagou Village, Li County, Sichuan; 22 Apr. 2023; Wei Han	male
	PseuSimiTZG	PQ059675	Tazigou, Parktou Township, Li County, Sichuan; 18 Apr. 2023; Wei Han	male
*P. yunnanensis*	PseuYunnTM	OP215869	Tongmai Town, Bomê, Tibet; 12 Aug. 2017; Jian-Yue Qiu, Hao Xu	male
	PseuYunnCY	OP215865	Zayü, Tibet; 14 Aug. 2015; Lu Qiu	male
	PseuYunnBM	OP215866	Bomê, Tibet; 11 Jul. 2016; Jian-Yue Qiu, Hao Xu	male
*P. baimaensis*	PseuBaim	PQ059685	Baima Village, Pingwu County, Sichuan; 4 Aug. 2019; Lu Qiu	male
*P. latizona*	PseuLatiSM	PQ059683	Caoke Village, Sichuan; 20 Jul. 2022; Wei Han, Xin-Xing Luo	female
*P. longiseta*	PseuLatiDB1	PQ059691	Danba County, Sichuan; 12 Jul. 2017; Jian-Yue Qiu, Hao Xu	male
	PseuLatiDB2	PQ059692	Jiaju Zangzhai, Danba County, Sichuan; 12 Jul. 2017; Jian-Yue Qiu, Hao Xu	male
	PseuLong1	PQ059684	Baima Snow Mountain, Yunnan; 27 Jul. 2020; Wei Han, Xin-Xing Luo, Lin Guo	female
*P. flava*	PseuFlav	PQ059689	Liude Village, Yunnan; 9 Jul. 2021; Lu Qiu, Hao Xu	female
*P. magna*	PseuMagn	PQ059688	Jinchuan County, Sichuan; 2020; Jian-Yue Qiu	male
*P. deficiens*	PseuDefiHS	PQ059687	Heishui County, Sichuan; 22 Jun. 2021; Lu Qiu, Hao Xu	nymph
*P. fusca*	PseuDefiCJS	PQ059686	Cuoji Mountain, Mao County, Sichuan; 6 Aug. 2019; Lu Qiu	female
	PseuFusc1	PQ059678	Cang Mountain, Yunnan; 29 Jul. 2022; Wei Han, Xin-Xing Luo	nymph
*P. dongi*	PseuDong	OP215872	Mt. Gaoligongshan, Baoshan, Yunnan; 13 Apr. 2017; Zhi-Wei Dong	male
*P. nigrinotum*	PseuNigr	OP215879	Mt. Jizushan, Bingchuan County, Yunnan; 20 Feb. 2016; Hao Xu, Jian-Yue Qiu	male
*P. wooi*	PseuWooi	OP215874	Mt. Ailaoshan, Xinping County, Yunnan; 11 May 2016; Lu Qiu	female
*P. daweishana*	PseuDawe	OP215877	Mt. Daweishan, Pinbian, Yunnan; 16 May 2016; Lu Qiu	nymph
*P. reducta*	PseuRedu	OP215886	Wadi Township, Mao County, Sichuan; 3 Oct. 2019; Hao Xu, Zhi-Teng Chen, Lu Qiu	nymph
*P. xuorum*	PseuXuor	OP215875	Caoke Township, Shimian County, Sichuan; 25 Aug. 2016; Hao Xu, Jian-Yue Qiu	male
**Outgroup**				
*Polyphaga plancyi*	PolyPlan	OQ736993	Xin County, Liaocheng City, Shandong; 31 Aug. 2015; Zhong Peng	male
*Eupolyphaga sinensis*	EupoSine	OP215846	Mt. Xishan, Beijing; 28 Apr. 2015; Bing-Qiang Wang	male

Photographs of the habitus and character details, including oothecae, were captured using a Canon R10 camera equipped with a Canon® RF-S18-150 mm F3.5-6.3 IS STM lens. Additional morphological features were imaged using a Leica M165C stereomicroscope and Leica Application Suite software. All images were processed using Adobe Photoshop CS6.

## ﻿Results

**Figure 1.**
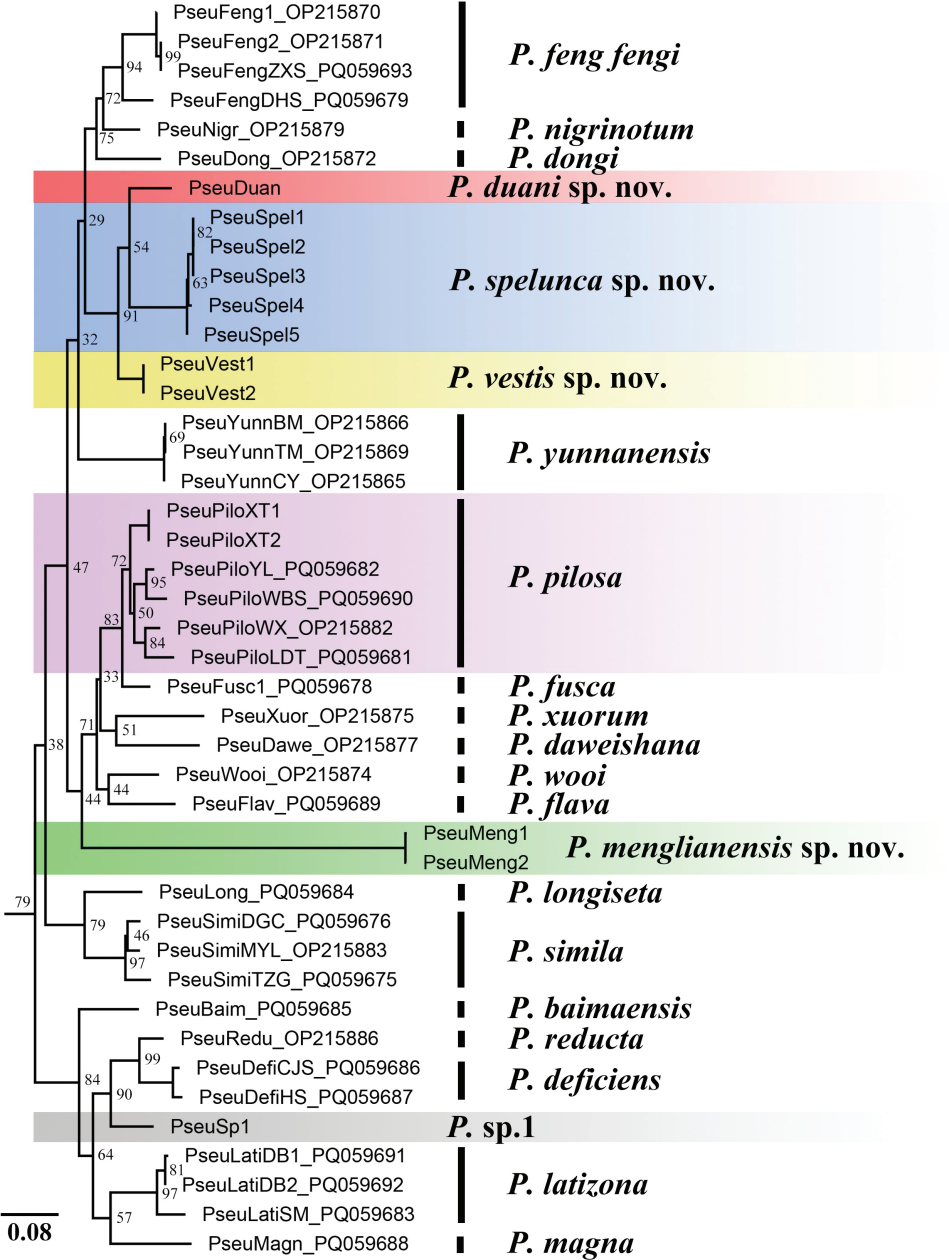
Phylogenetic tree of *Pseudoeupolyphaga* inferred by maximum-likelihood (ML) analysis of the mitochondrial COI fragment (outgroups not shown, and the newly added samples in this study are indicated by color blocks). UFBoot values are shown at the nodes.

Mitochondrial cytochrome *c* oxidase subunit I (COI) gene sequences of *Pseudoeupolyphaga* species were aligned to a consensus length of 660 bp. Intraspecific and interspecific genetic distances, computed using the Kimura 2-parameter (K2P) model, are provided in Suppl. material 1. Pairwise interspecific distances revealed considerable divergence among congeneric taxa, ranging from 5.80% (between *P. pilosa* (Qiu, Che & Wang, 2018) and *P. fusca*) to 28.25% (between *P. menglianensis* Ren & Han, sp. nov. and *P.* sp.1). The maximum-likelihood (ML) phylogenetic analysis of COI sequences, presented in Fig. 1, shows all morphological species as monophyletic groups. Nevertheless, most branches exhibited low bootstrap support values (< 95%), and several taxa were represented by single specimens, precluding rigorous statistical evaluation of their respective monophyly.

Examination of external morphology and male genitalia revealed four candidate new species within *Pseudoeupolyphaga*. Combined phylogenetic analyses and genetic divergence data further support the taxonomic status of these entities as new species, i.e., *P. duani* Ren & Han, sp. nov., *P. menglianensis* Ren & Han, sp. nov., *P. spelunca* Ren & Han, sp. nov., and *P. vestis* sp. nov. For more information on these new species, please refer to the Taxonomy section.

### ﻿Taxonomy

#### ﻿ Pseudoeupolyphaga duani

Taxon classificationAnimaliaBlattodeaCorydiidae

Ren & Han sp. nov.

04D0C3A3-51C6-5D26-8C90-A544C0E81A76

https://zoobank.org/9241BA50-DEDF-42F8-A000-F00128131EC1

Figs 2A–L, 8L

##### Type material.

***Holotype***: China • male (ZAFU); Yunnan Province, Pu’er City, Jinggu County; 1 Jun. 2025; Quan-Fu Duan leg; ZAFU-IC-200001. ***Paratypes***: China • 1 female & 2 nymphs (ZAFU); Luo-Jiang Liu leg; same collection data as holotype; ZAFU-IC-200002 to 200004 • 1 female & 2 nymphs (SWU); Luo-Jiang Liu leg; same collection data as holotype; SWU-B-CC-010074 to 010076.

##### Diagnosis.

The male tegmina maculae of this species most closely resemble those of *P. wooi* (Qiu, Che & Wang, 2018). However, in natural posture, the medial black maculae on both tegmina are fused in this species, and the abdomen (excluding the terminal segment) is light yellowish brown. In contrast, *P. wooi* exhibits non-fused medial black maculae on the tegmina in natural posture, with the abdomen (excluding the terminal segment) being dark yellowish brown. Furthermore, females of this species possess four symmetrical yellow maculae near the anterior margin on both the mesonotum and metanotum, whereas *P. wooi* females bear only two conspicuous symmetrical yellow maculae solely on the metanotum.

##### Description.

**Holotype. Measurements (mm).** Overall length (including tegmen): 26.27; body length: 17.82; body width (tegmina not included):8.46; tegmen length × width: 21.73 × 7.74; pronotum length × width: 7.42 × 4.85.

**Figure 2.**
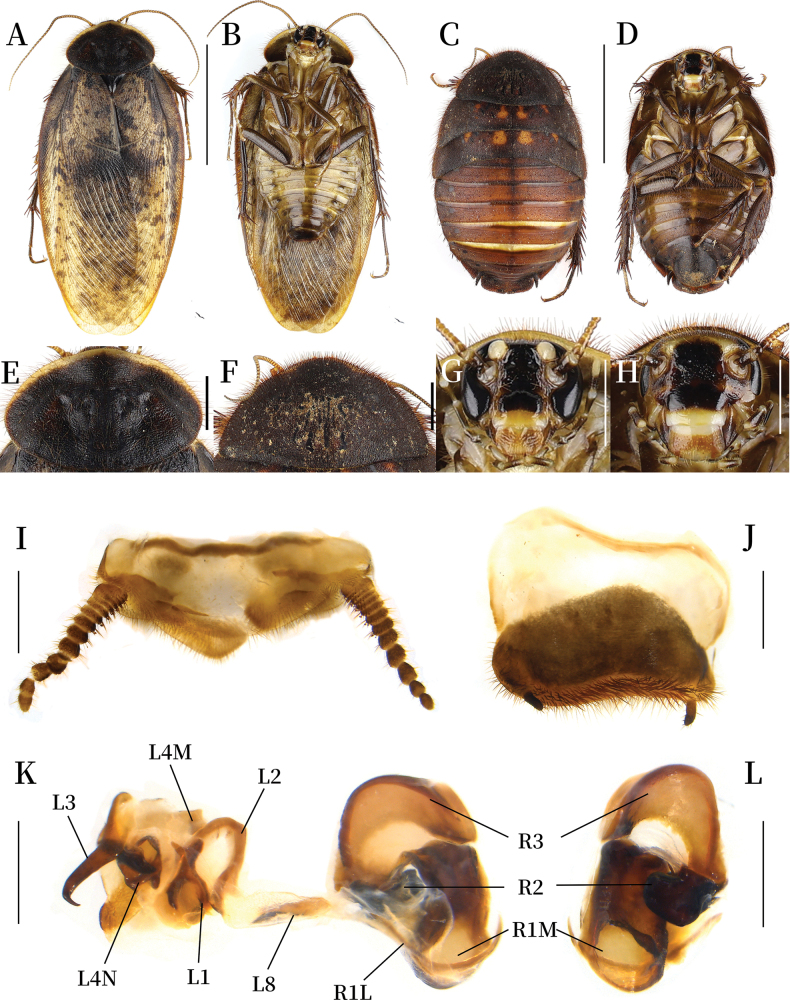
*Pseudoeupolyphaga duani* Ren & Han, sp. nov. **A, B, E, G, I–L.** Male holotype; **C, D, F, H.** Female paratype; **A.** Habitus, dorsal view; **B.** Habitus, ventral view; **C.** Habitus, dorsal view; **D.** Habitus, ventral view; **E.** Pronotum, dorsal view; **F.** Pronotum, dorsal view; **G.** Head, ventral view; **H.** Head, ventral view; **I.** Supra-anal plate, ventral view; **J.** Subgenital plate, ventral view; **K.** Genitalia, dorsal view; **L.** Right phallomere, right-ventral view. Scale bars: 1.0 cm (**A–D**); 0.2 cm (**E–H**); 0.1 cm (**I–L**).

**Coloration.** Pronotum black, anterior margin white. Tegmina and hind wings yellowish brown, maculae black (Fig. 2A, E). Eyes, vertex, post-clypeus and spaces between ocelli black. Ocelli and antennal sockets white. Ante-clypeus yellowish white. Antennae and labrum yellowish brown. Labial palpi brown (Fig. 2G). Legs black brown, spines yellowish brown to black. Pulvilli and arolia white. Sterna yellowish brown, margins and distal part black (Fig. 2B).

**Body. Head**: Sub-rounded, nearly completely hidden under pronotum. Eyes and ocelli well-developed. Interocular space narrower than the distance between ocelli, the latter narrower than the distance between antennal sockets (Fig. 2G). **Pronotum**: Oval-shaped, widest near the middle. Surface densely covered with short setae and long pubescence, central portion bearing symmetrical black stripes. Anterior whitish margin narrow, clearly delineated from black areas (Fig. 2E). **Tegmina and hind wings**: Densely covered with black maculae, markings on the basal lateral margins and mid-region more densely distributed than other areas (Fig. 2A). **Legs**: Slender, front femur Type C1. Pulvilli and arolia present (Fig. 2B). **Abdomen**: Smooth. Supra-anal plate transverse, pubescent, posterior margin protruded medially. Paraprocts simple (Fig. 2I). Subgenital plate with short setae, hind margin slightly asymmetric. Styli thin and long (Fig. 2J). **Genitalia**: Well-sclerotized. L1 apically bearing two short branches; the two basal branches distinct and elongated. L2 arcuately curved. Genital hook (L3) medially swollen, tapering toward hooked apex. L4M broadly lamellate. L4N well-developed; pda and paa strongly curved. L8 irregular, narrow and plate-like. R1M stoutly expanded terminally. R1L elongate and banded. R2 divided into two chunks. R3 broadly concave (Fig. 2K, L).

**Female paratype (mm).** Body length: 20.54; body width: 12.02; pronotum length × width: 8.91 × 5.58.

**Coloration.** Terga reddish brown to blackish brown, with four symmetrical yellow maculae at the anterior margin of the meso- and metanotum (Fig. 2C). Pronotum dark reddish brown, pubescence light reddish brown (Fig. 2F). Vertex, eyes, space between ocelli and post-clypeus black. Antennal sockets white. Antennae yellowish brown. Ocelli, ante-clypeus, and basal part of labrum pale yellow. Middle and distal part of labrum yellowish brown. Legs dark brown, spines reddish brown to black. Sterna brown to yellowish brown. Subgenital plate black (Fig. 2D, H).

**Body.** The widest point of pronotum near the hind margin. Anterior whitish margin absent, bearing symmetrical black stripe medially (Fig. 2F). Ocelli degraded to two spots. Interocular space almost equal to the distance between antennal sockets, both wider than the distance between ocelli. Front femur Type C1. Arolia and pulvilli absent (Fig. 2D, H).

**Nymph.** Pronotum also with four symmetrical yellow maculae, the rest similar to the female.

**Ootheca.** Unknown.

##### Etymology.

This species is named after the collector, Mr Quan-Fu Duan, in recognition of his contribution to its discovery.

##### Remarks.

This species exhibits the smallest genetic distance (8.75%) to *P. vestis*, while distances to all other congeners exceed 9%. The male forewing maculae and the four distinct maculae on the female mesonotum and metanotum readily distinguish this species from other members of the genus, providing robust morphological support for its establishment as a new species.

#### ﻿ Pseudoeupolyphaga menglianensis

Taxon classificationAnimaliaBlattodeaCorydiidae

Ren & Han sp. nov.

F7002A18-1F5E-5E71-9F73-9A102C1B1695

https://zoobank.org/A12D88FC-0F06-4265-A434-EE7BD1F76E43

**Figure 3.**
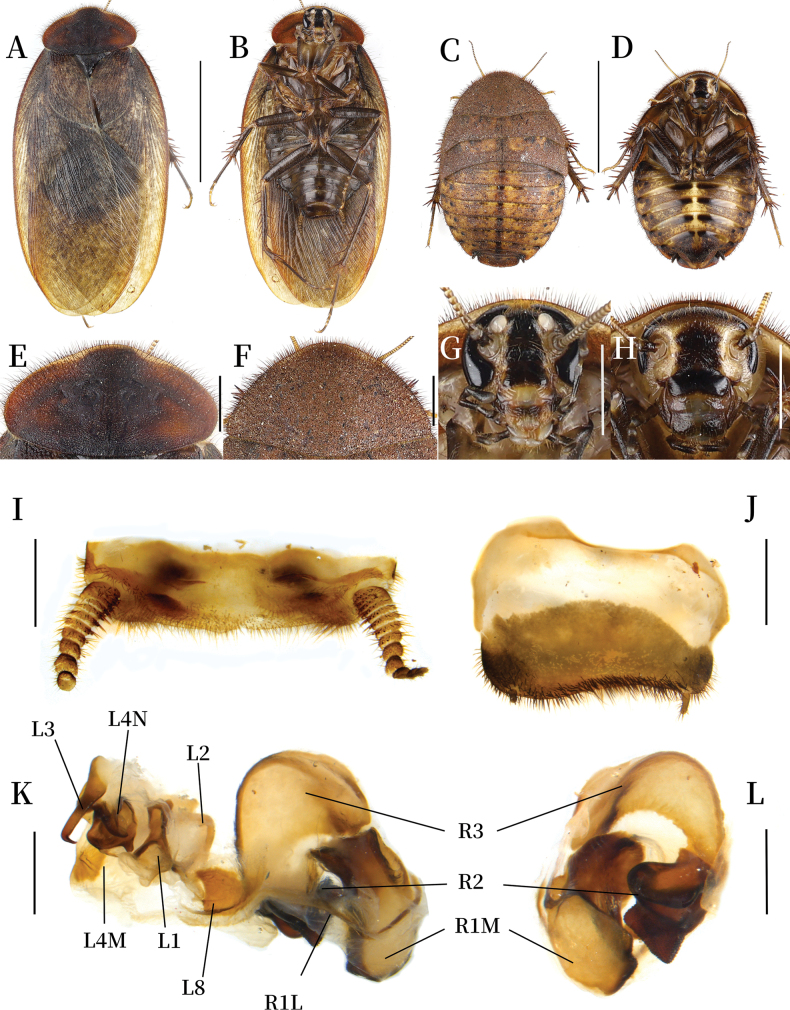
*Pseudoeupolyphaga menglianensis* Ren & Han, sp. nov. **A, B, E, G, I–L.** Male holotype; **C, D, F, H.** Female paratype; **A.** Habitus, dorsal view; **B.** Habitus, ventral view; **C.** Habitus, dorsal view; **D.** Habitus, ventral view; **E.** Pronotum, dorsal view; **F.** Pronotum, dorsal view; **G.** Head, ventral view; **H.** Head, ventral view; **I.** Supra-anal plate, ventral view; **J.** Subgenital plate, ventral view; **K.** Genitalia, dorsal view; **L.** Right phallomere, right-ventral view. Scale bars: 1.0 cm (**A–D**); 0.2 cm (**E–H**); 0.1 cm (**I–L**).

Figs 3A–L, 8K, 10A, B, D–G

##### Type material.

***Holotype***: China • male (ZAFU); Yunnan Province, Pu’er City, Menglian County, Nayun Town, Mengwai Village; 6 May, 2025; Zhong-Hong Luo leg; ZAFU-IC-200005. ***Paratypes***: China • 1 female (pre-adult) & 4 nymphs (ZAFU); same collection data as holotype; ZAFU-IC-200006 to 200010 • 1 male & 2 nymphs (SWU); same collection data as holotype; SWU-B-CC-010077 to 010079.

##### Diagnosis.

This species exhibits highly similar tegmina maculae in males and dorsal maculae in females to those of *P. daweishana*. The primary diagnostic differences are: (1) Males of this species possess a light yellowish-brown abdomen, whereas *P. daweishana* males have a nearly uniform black abdomen; 2) The anterior whitish margin on the male pronotum is extremely narrow in this species, contrasting with the significantly wider counterpart in *P. daweishana* males; and (3) The sclerite paa of this species is broad with a short finger-like projection at the right posterior margin, while in *P. daweishana*, the paa bears a long finger-like projection.

##### Description.

**Holotype. Measurements (mm).** Overall length (including tegmen): 25.63; body length: 17.26; body width (tegmina not included):8.75; tegmen length × width: 23.02 × 8.62; pronotum length × width: 7.73 × 4.64.

**Coloration.** Body dark brown (Fig. 3A, B). Pronotum reddish brown, with yellowish-brown pubescence along the outer margin, anterior margin partially yellow (Fig. 3E). Tegmina and hind wings yellowish brown, with dark brown maculae (Fig. 3A, B). Vertex, eyes, and spaces between ocelli black. Ocelli and antennal sockets white. Antennae yellow, ante-clypeus pale yellow, post-clypeus black. Labrum yellowish brown, middle part dark brown (Fig. 3G). Legs dark brown, spines yellowish brown to black. Pulvilli and arolia white. Sterna dark brown (Fig. 3B).

**Figure 4.**
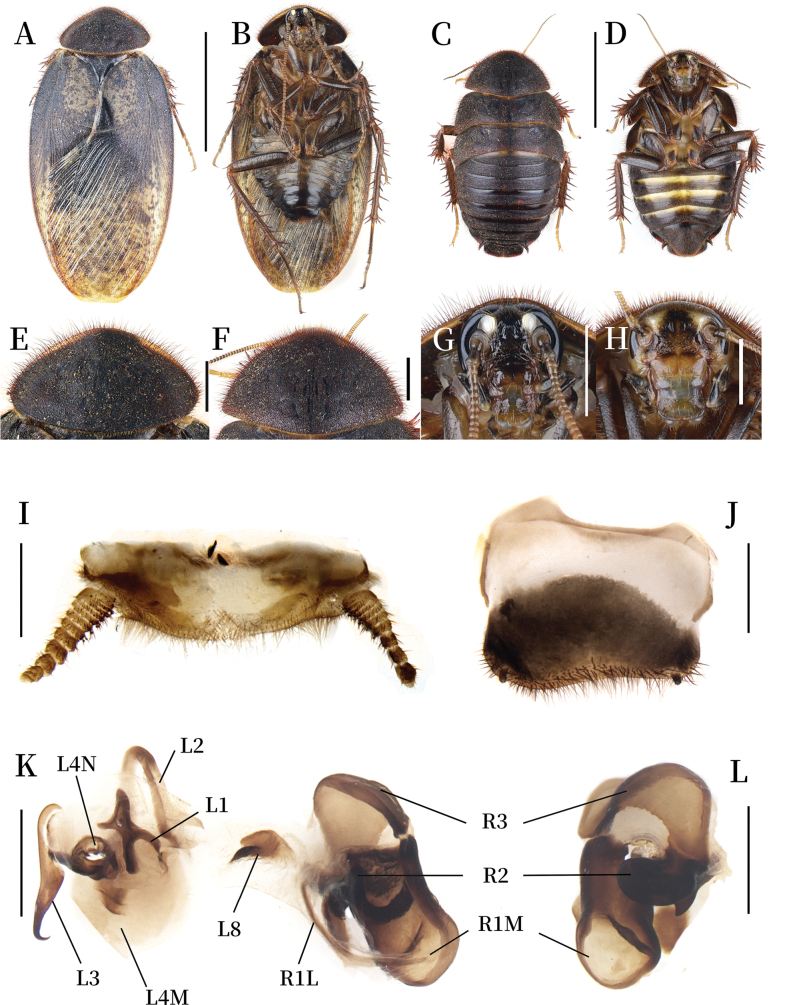
*Pseudoeupolyphaga spelunca* Ren & Han, sp. nov. **A, B, E, G, I–L.** Male holotype; **C, D, F, H.** Female paratype; **A.** Habitus, dorsal view; **B.** Habitus, ventral view; **C.** Habitus, dorsal view; **D.** Habitus, ventral view; **E.** Pronotum, dorsal view; **F.** Pronotum, dorsal view; **G.** Head, ventral view; **H.** Head, ventral view; **I.** Supra-anal plate, ventral view; **J.** Subgenital plate, ventral view; **K.** Genitalia, dorsal view; **L.** Right phallomere, right-ventral view. Scale bars: 1.0 cm (**A–D**); 0.2 cm (**E–H**); 0.1 cm (**I–L**).

**Body. Head**: Sub-rounded, almost entirely hidden under pronotum. Eyes and ocelli well-developed. Ocelli ridge indistinct, bearing a row of slender setae along the upper edge. Interocular space narrower than the distance between ocelli, the latter narrower than the distance between antennal sockets (Fig. 4G). **Pronotum**: Oval-shaped, widest near the middle. Surface densely covered with short setae and long pubescence, with symmetrical black stripes medially. Anterior whitish margin extremely narrow, medially almost interrupted (Fig. 3E). **Tegmina and hind wings**: Tegmina densely covered with small and diffuse maculae; maculae at basal and medial regions more densely distributed than at apical region (Fig. 3A, B). **Legs**: Slender, front femur Type C1. Pulvilli and arolia present (Fig. 3B). **Abdomen**: Smooth, light yellowish brown. Supra-anal plate narrow, distinctly pubescent. Cerci slender. Subgenital plate slightly asymmetrical. Styli long. **Genitalia**: Well-sclerotized. L1 apically swollen and bearing one short branch that pointed to the top right corner; the two basal branches thin and elongated. L2 arcuately curved. Genital hook (L3) straight and short. L4M broadly lamellate. L4N well-developed; pda narrow, paa wide and with a finger-like protrusion at the right bottom. L8 plate-like. R1M stoutly expanded terminally. R1L elongate and thin. R2 divided into two chunks. R3 broadly concave (Fig. 3K, L).

**Female (pre-adult) paratype(mm).** Body length: 17.72; body width: 10.56; pronotum length × width: 8.23 × 4.90.

**Coloration.** Terga yellow to brownish yellow, densely covered with brownish-yellow maculae, median with a black-brown longitudinal line (Fig. 3C). Pronotum brownish yellow, densely covered with short setae (Fig. 3F). Vertex, eyes, and the space between ocelli and post-clypeus are black. Antennal sockets and ante-clypeus white. Ocelli and the areas surrounding the ocelli and antennal sockets yellowish white. Antennae yellowish brown. Labrum blackish brown. Legs blackish brown, with spines ranging from yellowish brown to black. Sterna yellow, densely covered with brownish-yellow maculae, median with a wide longitudinal yellow line. Middle part of subgenital plate black (Fig. 3D, H).

**Body.** The widest point of pronotum near the hind margin. Anterior whitish margin absent (Fig. 3F). Ocelli degraded to two spots. Interocular space almost equal to the distance between antennal sockets, both wider than the distance between ocelli. Front femur Type C1. Arolia and pulvilli absent (Fig. 3D, H).

**Female.** Unknown

**Nymph.** Similar to the pre-adult female.

**Ootheca.** Unknown.

##### Etymology.

The name of this species comes from the type locality, Menglian County, Pu’er City, Yunnan Province.

##### Remarks.

Intraspecific genetic distance between two specimens of this species is 0.31%, while its minimum distance to other congeners exceeds 20%. The geographical distance between this species and several other species also found in Pu’er City is relatively large (exceeding 100 km). Although morphologically similar to *P. daweishana*, these taxa exhibit diagnostic differences in: (1) male abdominal coloration, (2) width of the anterior whitish margin on the pronotum, and (3) structure of the sclerite paa. The substantial genetic divergence (21.9%) and allopatric distribution (>400 km straight-line distance) further support their recognition as distinct species.

#### ﻿ Pseudoeupolyphaga spelunca

Taxon classificationAnimaliaBlattodeaCorydiidae

Ren & Han sp. nov.

80CD9127-DC8D-5216-95E6-30C195288935

https://zoobank.org/45EEB992-CDC7-4ED2-B9B9-F1D0529AB317

Figs 4A–L, 8B, E, G, H

##### Type material.

***Holotype***: China • male (ZAFU); Yunnan Province, Pu’er City, Yunxian Township, Dashitou Village; 19 Dec. 2024; Hang Qiu leg; ZAFU-IC-200011. ***Paratypes***: China • 1 female & 5 nymphs (ZAFU); same collection data as holotype; ZAFU-IC-200012 to 200017 • 1 male & 2 females & 2 nymphs (SWU); same collection data as holotype; SWU-B-CC-010080 to 010084.

##### Diagnosis.

The males of this species resemble certain *Pseudoeupolyphaga* congeners with densely maculate tegmina, such as *Pseudoeupolyphaga daweishana* (Qiu, Che & Wang, 2018), *Pseudoeupolyphaga hengduana* (Woo & Feng, 1992), and *Pseudoeupolyphaga fengi yongshengensis* (Qiu, Che & Wang, 2018). However, it is readily distinguished from *P. daweishana* and *P. hengduana* by the conspicuously sparser maculae in the anal field of the tegmina compared to adjacent areas. Furthermore, it differs from *P. fengi yongshengensis* by the absence of a longitudinal yellow stripe on the mid-ventral sternum—a diagnostic trait presents in the latter. Additional key diagnostic characters include: denser maculae on the tegmina of *P. spelunca* males, concentrated primarily basally and medially, but sparse apically. In contrast, the three congeneric males exhibit more uniformly distributed tegmina maculae without such pronounced regional differentiation.

##### Description.

**Holotype. Measurements (mm).** Overall length (including tegmen): 24.68; body length: 18.74; body width (tegmina not included): 9.40; tegmen length × width: 20.83 × 8.58; pronotum length × width: 7.73 × 4.14.

**Coloration.** Body dark brown (Fig. 4A, B). Pronotum black, outer margin with pale yellow pubescence, anterior margin yellow (Fig. 4E). Tegmina yellowish brown, with large dark brown patches at the basal and middle region; remaining areas with evenly distributed dark brown markings. Hind wings pale yellow, bearing dark brown plaques (Fig. 4A, B). Face black. Antennae yellow. Eyes black. Ocelli white. Ante-clypeus pale yellow, post-clypeus dark brown. Labrum yellow (Fig. 4G). Legs dark brown. Pulvilli and arolia white. Abdomen dark brown, gradually deepening in color toward the lateral and distal end (Fig. 4B).

**Body. Head**: Sub-rounded, hidden under pronotum. Eyes and ocelli well-developed. Ocellar ridge slightly curved, bearing a row of slender setae along the upper edge. Interocular space narrower than the distance between ocelli, the latter narrower than the distance between antennal sockets. Clypeus developed (Fig. 4G). **Pronotum**: Oval-shaped, widest near the middle. Surface densely covered with short setae and long pubescence, central portion bearing symmetrical black stripes. Anterior whitish margin extremely narrow, disconnect at the middle of the front margin (Fig. 4E). **Tegmina and hind wings**: Basal half of tegmina nearly completely covered by black patches except anal area. Anal area and distal half of tegmina bearing variably sized markings (Fig. 4A, B). **Legs**: Slender, front femur Type C1. Pulvilli and arolia present (Fig. 4B). **Abdomen**: Supra-anal plate transverse, pubescent, posterior margin slightly protruded medially. Paraprocts simple (Fig. 4I). Subgenital plate with short setae, hind margin concave in the middle, the left side less prominent than the right side. Styli short and small (Fig. 4J). **Genitalia**: Well-sclerotized. L1 apically swollen and bearing two short branches; the two basal branches distinct and elongated. L2 arcuately curved. Genital hook (L3) medially swollen, tapering toward hooked apex. L4M broadly lamellate. L4N well-developed, pda and paa strongly curved. L8 irregular, plate-like. R1M stoutly expanded terminally. R1L elongate and banded. R2 divided into two chunks. R3 broadly concave (Fig. 4K, L).

**Female paratype (mm).** Body length: 21.92; body width: 12.05; pronotum length × width: 8.64 × 4.87.

**Coloration.** Terga deep reddish brown (Fig. 4C). Vertex black. Face yellow. Antennae yellow. Ocelli yellowish white, space between ocelli, antennal sockets and post-clypeus brown to dark brown. Ante-clypeus and labrum pale yellowish brown. Post-clypeus yellowish brown (Fig. 4H). Legs dark brown overall, tarsus slight pale. Spines on legs yellowish brown, terminal nearly black. Sterna uniformly dark brown and middle part with yellow strips (Fig. 4D).

**Body.** Pronotum widest near the hind margin, middle part with symmetrical black dark stripes, anterior whitish margin indistinct (Fig. 4F). Ocelli big but not prominent, presenting white spots in a near-triangular shape. The distance between ocelli narrower than the distance between antennal sockets, both measurements less than interocular space (Fig. 4H). Front femur Type C1. Arolia and pulvilli absent.

**Nymph.** Similar to the female.

**Ootheca.** Reddish brown, surface with parallel and dense longitudinal lines. Ridges of serrated protuberances slightly small. No respiratory canals (Fig. 8B, E).

##### Etymology.

The species epithet is derived from the Latin term *spelunc*, which refers to natural caves, indicating it was found in dark subterranean environments (a few hundred meters from the cave entrance).

##### Remarks.

Intraspecific genetic divergence among five specimens of this species ranges from 0% to 1.08% (all specimens from Dashitou Village, Yunxian Township). It exhibits the closest genetic proximity to *P. vestis* (7.75%–8.93%), followed by *P. duani* (9.71%–10.95%), while distances to all other congeners exceed 10%. The male tegmina maculae provide diagnostically distinct characters that readily differentiate this species from both *P. vestis* and *P. duani*.

#### ﻿ Pseudoeupolyphaga vestis

Taxon classificationAnimaliaBlattodeaCorydiidae

Ren & Han sp. nov.

D1C8B03A-B7B3-5737-B7A1-00E83BE1B155

https://zoobank.org/1DB01996-1C9F-4524-BBE1-59383DA7CA91

**Figure 5.**
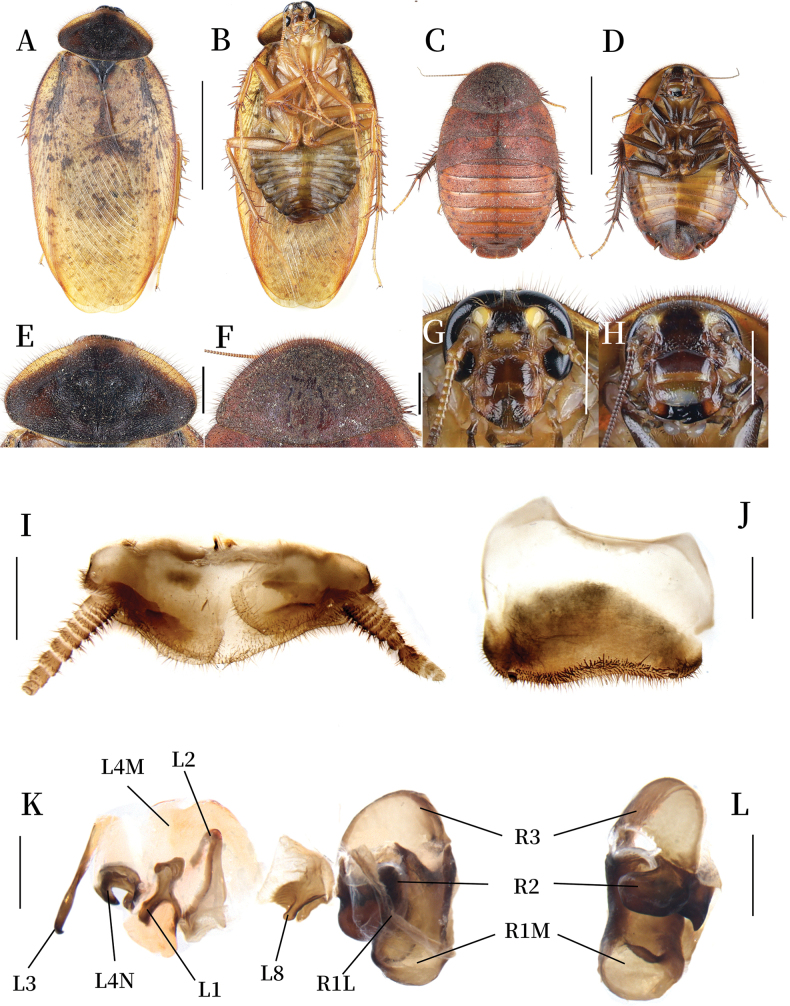
*Pseudoeupolyphaga vestis* Ren & Han, sp. nov. **A, B, E, G, I–L.** Male holotype; **C, D, F, H.** Female paratype; **A.** Habitus, dorsal view; **B.** Habitus, ventral view; **C.** Habitus, dorsal view; **D.** Habitus, ventral view; **E.** Pronotum, dorsal view; **F.** Pronotum, dorsal view; **G.** Head, ventral view; **H.** Head, ventral view; **I.** Supra-anal plate, ventral view; **J.** Subgenital plate, ventral view; **K.** Genitalia, dorsal view; **L.** Right phallomere, right-ventral view. Scale bars: 1.0 cm (**A–D**); 0.2 cm (**E–H**); 0.1 cm (**I–L**).

Figs 5A–L, 8A, D, I, J

##### Type material.

***Holotype***: China • male (ZAFU); Yunnan Province, Pu’er City, Liushun Town; 25 Dec. 2024; Quan-fu Duan leg; ZAFU-IC-200018. ***Paratypes***: China • 1 female & 1 nymph (ZAFU); same collection data as holotype; ZAFU-IC-200019 to 200020 • 1 female & 1 nymph (SWU); same collection data as holotype; SWU-B-CC-010085 to 010086.

##### Diagnosis.

The male of this species resembles *Pseudoeupolyphaga deficiens* Han, Che & Wang, 2024, but differs in having a narrower anterior whitish margin in the pronotum. In addition, the abdominal tergites of females are uniformly orange-red (observation of two samples), making it easy to distinguish from most congeners.

##### Description.

**Holotype. Measurements (mm).** Overall length (including tegmen): 26.30; body length: 19.48; body width (tegmina not included): 10.17; tegmen length × width: 22.77 × 9.30; pronotum length × width: 8.63 × 4.06.

**Coloration.** Pronotum deep reddish brown, setae yellowish, anterior whitish margin yellow (Fig. 5A, E). Tegmina pale yellow, interspersed with varying-sized blackish-brown maculae (Fig. 5A). Eyes, vertex, and space between ocelli black. Ocelli pale yellow. Antennal sockets, and ante-clypeus yellowish brown. Post-clypeus, labrum, labial palpi and maxillary palpi blackish brown (Fig. 5G). Legs yellowish brown, with spines ranging from reddish brown to black. Pulvilli and arolia white. Sterna pale yellow, middle and distal part gray to pale black (Fig. 5B).

**Body. Head**: Sub-rounded. Eyes and ocelli well-developed. Interocular space narrower than the distance between ocelli, the latter narrower than the distance between antennal sockets. Clypeus developed (Fig. 5G). **Pronotum**: Elliptical, widest near the posterior margin. Surface densely covered with short setae, bearing symmetrical black stripe medially. Anterior whitish margin clearly delineated from deep reddish-brown areas (Fig. 5E). **Tegmina and hind wings**: Tegmina with dark brown patch confluent basally along outer margin. Remaining areas sparsely scattered with variably sized blackish- brown maculae (Fig. 5A). **Legs**: Slender, front femur Type C1. Pulvilli and arolia present (Fig. 5B). **Abdomen**: Supra-anal plate transverse, pubescent, posterior margin protruded medially. Paraprocts simple (Fig. 5I). Subgenital plate bearing short setae, posterior margin slightly curved. Styli short and small (Fig. 5J). **Genitalia**: Well-sclerotized. L1 apically dilated, basally bifurcated. L2 arched curved, distally dilated. Genital hook (L3) thin and straight, curved hook section small. L4M broad lamellate. L4N developed, pda and paa broad and curved. L8 irregular, lamellate, bearing a long clavate process. R1M stoutly expanded terminally. R1L elongate and banded. R2 divided into two chunks, narrowly spaced, with rounded margins. R3 broadly concave (Fig. 5K, L).

**Paratype. Measurements (mm).** Body length: 20.08; body width: 12.43; pronotum length × width: 9.14 × 5.28.

**Coloration.** Terga orange-red (Fig. 5C). Head dark brown. Antennae yellow. Ocelli white. Ante-clypeus yellowish brown. Post-clypeus dark brown. Labrum with a white patch basally, remaining portions yellowish brown (Fig. 5H). Legs reddish brown. Spines on the leg reddish brown, terminal nearly black. Sterna yellowish brown to orange-red, subgenital plate black (Fig. 5D).

**Body.** The widest point of pronotum near the hind margin, middle part with symmetrical dark-black stripe, anterior yellow margin indistinct (Fig. 5F). Ocelli indistinct, degenerated to two patches. The distance between ocelli narrower than the distance between antennal sockets, and the latter narrower than interocular space (Fig. 5H). Front femur Type C1. Arolia and pulvilli absent.

**Nymph.** Similar to female, only the coloration is slightly paler.

**Ootheca.** Reddish brown, surface with many parallel, dense longitudinal lines. Ridges of serrated protuberances with broad bases, slightly curved lateral edges and blunt tips. No respiratory canals (Fig. 8A, D).

##### Etymology.

The species epithet is derived from the Latin word *vest*, denoting its tegmina markings resembling a vest.

##### Remarks.

This species exhibits relatively close genetic divergence to both *P. spelunca* (7.75%–8.93%) and *P. duani* (8.75%). Morphologically, the male tegmina maculae of this species closely resemble those of *P. deficiens*, and females of both taxa share a tergum pigmentation tending toward orange-red. However, the former displays significantly deeper pigmentation, and this species exhibits a pronounced color difference between the terga and sterna (terga orange-red, sterna yellowish brown to orange-red), whereas in *P. deficiens*, the pigmentation of these surfaces is largely consistent. In addition, the substantial genetic divergence between *P. vestis* and *P. deficiens* reaches 17.53%, supporting their recognition as distinct species.

#### ﻿ Pseudoeupolyphaga pilosa

Taxon classificationAnimaliaBlattodeaCorydiidae

(Qiu, Che & Wang, 2018)

74819DD9-52CD-5889-986B-6D27AEED1DBF

**Figure 6.**
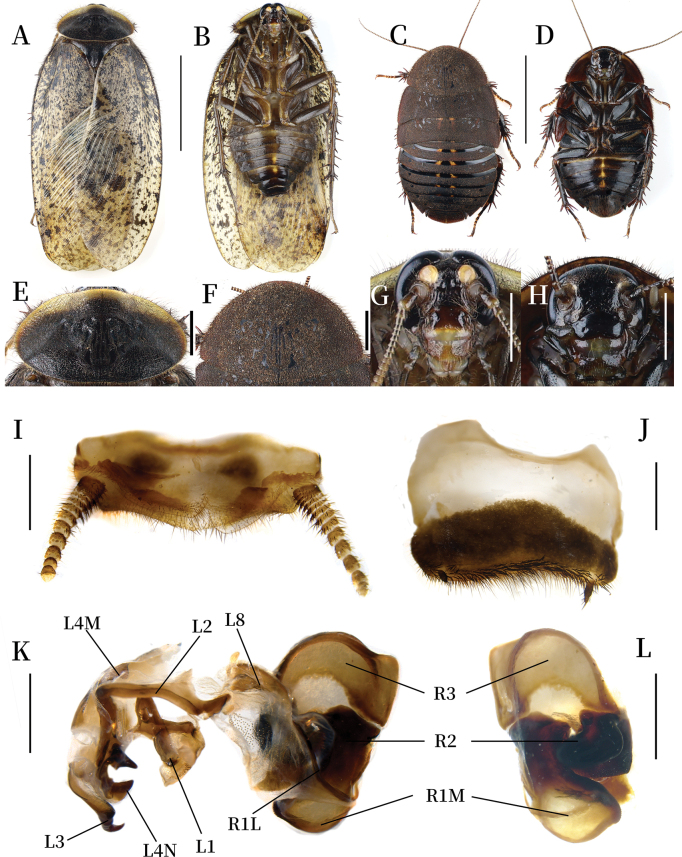
*Pseudoeupolyphaga pilosa*. **A, B, E, G, I–L.** Male holotype; **C, D, F, H.** Female paratype; **A.** Habitus, dorsal view; **B.** Habitus, ventral view; **C.** Habitus, dorsal view; **D.** Habitus, ventral view; **E.** Pronotum, dorsal view; **F.** Pronotum, dorsal view; **G.** Head, ventral view; **H.** Head, ventral view; **I.** Supra-anal plate, ventral view; **J.** Subgenital plate, ventral view; **K.** Genitalia, dorsal view; **L.** Right phallomere, right-ventral view. Scale bars: 1.0 cm (**A–D**); 0.2 cm (**E–H**); 0.1 cm (**I–L**).

Figs 6A–L, 8C, F, 9A–E

##### Type locality.

“Yunnan Province, Diqing Prefecture, Weixi County, Pantiange Township, A valley near Zhazi; 2970 m”.

##### New material examined.

China • 1 male, 1 female & 8 nymphs (ZAFU); Yunnan Province, Dali Bai Autonomous Prefecture, Jianchuan County, Xiangtu Township; 15 Apr. 2025; He Zhang leg; ZAFU-IC-200021 to 200030 • 1 male & 1 female (SWU); Yunnan Province, Dali Bai Autonomous Prefecture, Jianchuan County, Xiangtu Township; 15 Apr. 2025; He Zhang leg; SWU-B-CC-010087 to 010088.

##### Remarks.

The male specimen collected in Jianchuan County exhibits dense tegmina markings similar to those of *P. pilosa* and *P. longiseta*. However, both sexes of this population possess a distinct longitudinal yellow line medially on their abdominal sternites, a characteristic allowing their immediate distinction from *P. longiseta*. The tegmina markings pattern of the Jianchuan male, however, is noticeably denser than that of the *P. pilosa* holotype (closer to specimens from Blue Moon Valley, Yulong Snow Mountain) and exhibits a significantly greater body length (excluding wings: 20.3 mm vs. 15.6–16.8 mm in the type locality). Given the minimal genetic divergence from other *P. pilosa* populations (5.13–6.90%) and proximity to its known distribution, we tentatively assign these Jianchuan specimens to *P. pilosa* in the present study.

#### ﻿ Pseudoeupolyphaga

Taxon classificationAnimaliaBlattodeaCorydiidae

sp. 1

D92A8818-10A9-58AB-9BEE-D66914A22292

Figs 7A–D, 10C

##### Material examined.

China • 1 female (ZAFU); Sichuan Province, Ganzi Tibetan Autonomous Prefecture, Luding County; 27 Mar. 2025; Ding Zhou leg; ZAFU-IC-200031 • 1 female & 2 nymphs (SWU), Sichuan Province, Ganzi Tibetan Autonomous, Luding County; 27 Mar. 2025; Ding Zhou leg; SWU-B-CC-010089 to 010091.

##### Remarks.

In the phylogenetic tree, this species forms a sister group to *Pseudoeupolyphaga deficiens* Han, Che & Wang, 2024 + *Pseudoeupolyphaga reducta* (Qiu, 2022). Genetic distance analysis indicates that the specimen exhibits the smallest genetic distance (9.00%) to *P. reducta*, while its distance to *P. latizona* from Shimian County (adjacent to Luding County) is 16.42% and its distance to *P. deficiens* is 11.92–12.60%. No previous specimens of this genus have been collected from Luding County. However, considering the genetic distances, this specimen likely represents a new species. Further research is warranted upon the discovery of male specimens for comprehensive study.

**Figure 7.**
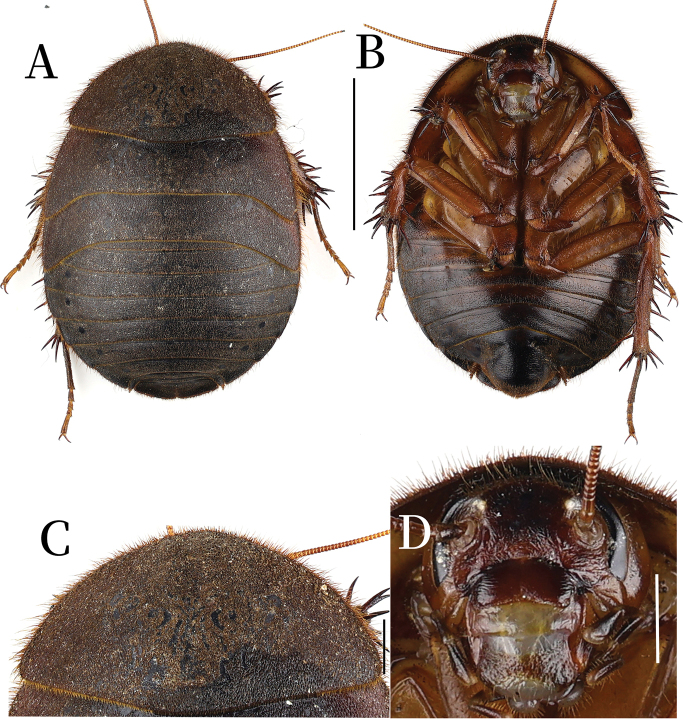
**A–D.***Pseudoeupolyphaga* sp. 1; **A.** Habitus, dorsal view; **B.** Habitus, ventral view; **C.** Pronotum, dorsal view; **D.** Head, ventral view. Scale bars: 1.0 cm (**A, B**); 0.2 cm (**C, D**).

**Figure 8.**
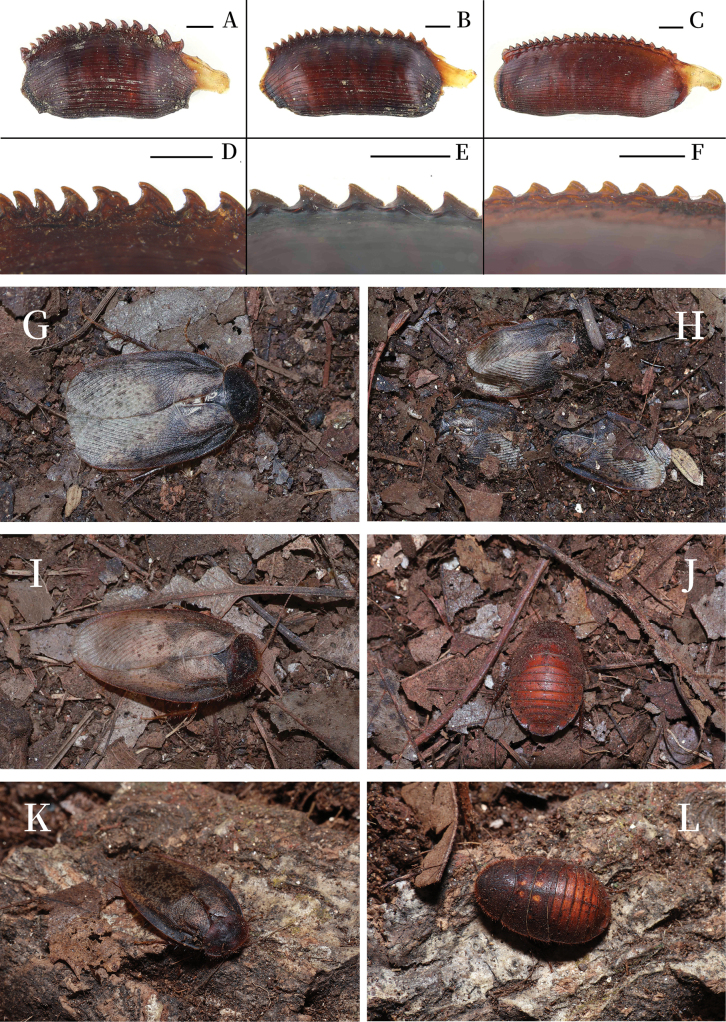
Oothecae of *Pseudoeupolyphaga* and living individuals. Oothecae lateral view (**A–C**) and close-up view to show the serrations (**D–F**). **A, D.***P. vestis* sp. nov.; **B, E.***P. spelunca* sp. nov.; **C, F.***P. Pilosa*; **G, H.** Male adults of *P. spelunca* in the laboratory; **I.** Male adults of *P. vestis* under the laboratory; **J.** Female adults of *P. vestis* under laboratory conditions; **K.** Male adults of *P. menglianensis* under the laboratory; **L.** Female adults of *P. dauni* under laboratory conditions (**G–L** by Yi-Ming Ren). Scale bars: 0.1 cm (**A-F**).

**Figure 9.**
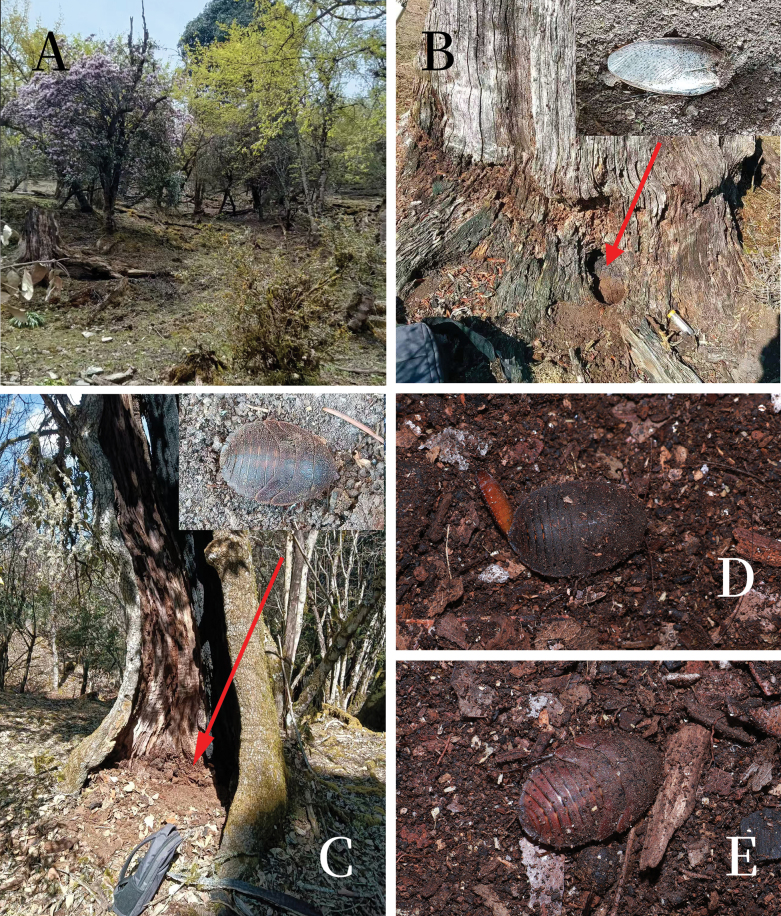
Habitats and living individuals of *Pseudoeupolyphaga pilosa* from Xiangtu Township, Jianchuan County, Dali Bai Autonomous Prefecture. **A.** Habitats of *P. pilosa*; **B, C.** Individuals collected under a tree (arrow points to the location where the insect was discovered); **D.** Female with an ootheca under lab conditions; **E.** Male sub-adults under laboratory conditions (**A–C** by He Zhang; **D–E** by Yi-Ming Ren).

**Figure 10.**
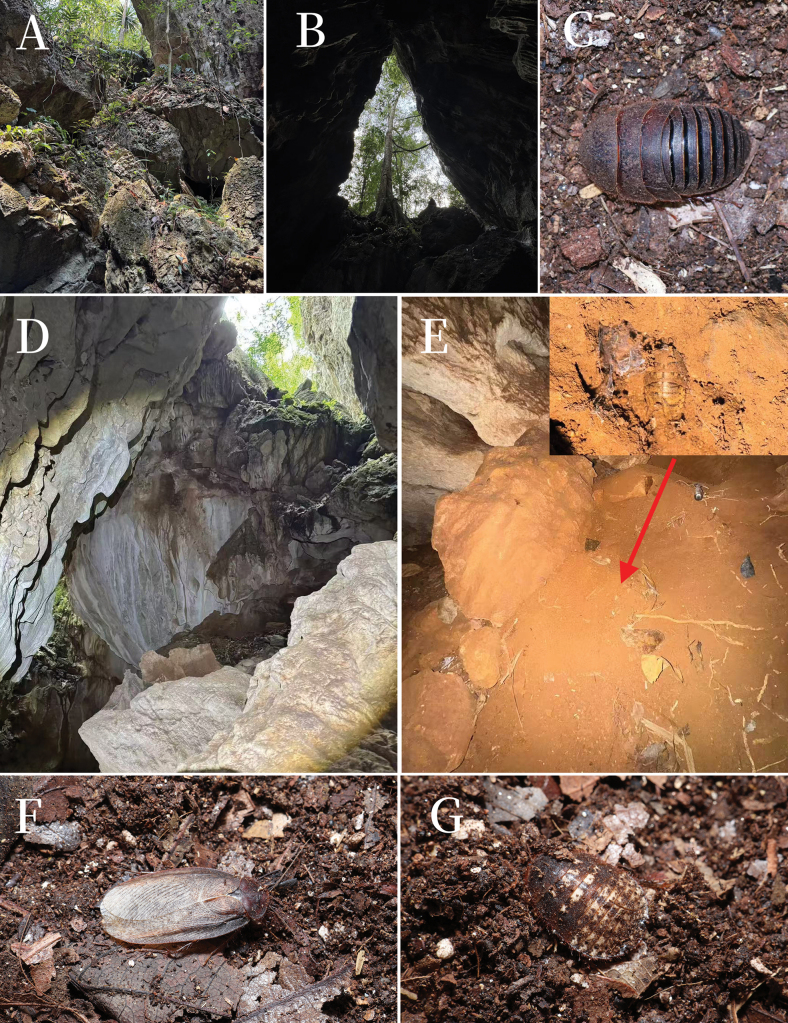
Habitats and living individuals of *Pseudoeupolyphaga* species. **A, B, D–G.** Photos of *P. menglianensis* sp. nov. from Yunnan Province, Pu’er City, Menglian Country, Nayun Town, Mengwai Group; **A, B, D.** Habitats of *P. menglianensis*; **C.***P.* sp. 1: female under lab conditions; **E.** Individuals collected from the soil (arrow points to the location where the insect was discovered); **F.** Males reared from the nymphs under lab conditions; **G.** Female nymph that has recently been exuviated under laboratory conditions (**A, B, D–E** by Mr Zhong-Hong Luo; **C, E–G** by Yi-Ming Ren).

## Supplementary Material

XML Treatment for Pseudoeupolyphaga duani

XML Treatment for Pseudoeupolyphaga menglianensis

XML Treatment for Pseudoeupolyphaga spelunca

XML Treatment for Pseudoeupolyphaga vestis

XML Treatment for Pseudoeupolyphaga pilosa

XML Treatment for Pseudoeupolyphaga

## ﻿Additional information

### Use of AI

No use of AI was reported.

### Author ORCIDs

Yi-Ming Ren https://orcid.org/0009-0003-1055-1236

Wei Han https://orcid.org/0000-0002-7243-1657

Yan-Li Che https://orcid.org/0000-0003-3214-9494

Ji-Rui Wang https://orcid.org/0000-0002-8388-1565

### Data availability

All of the data that support the findings of this study are available in the main text or Supplementary Information.

## Supplementary materials

Supplementary material 1 Gene sequences Data type xls Explanation note Intraspecific and interspecific genetic distances of *Pseudoeupolyphaga* species, computed using the Kimura 2-parameter (K2P) model. https://binary.pensoft.net/file/1482633This dataset is made available under the Open Database License (http://opendatacommons.org/licenses/odbl/1.0/). The Open Database License (ODbL) is a license agreement intended to allow users to freely share, modify, and use this Dataset while maintaining this same freedom for others, provided that the original source and author(s) are credited.Yi-Ming Ren, Wei Han, Yan-Li Che, Ji-Rui Wang

## References

[B1] Chopard L (1929) Orthoptera palaearctica critica VII. Les Polyphagiens de la faune paléarctique (Orth., Blatt.). Eos (Washington, D.C.) 5: 223–358.

[B2] Han W Qiu L Zhu J Wang ZQ Che YL (2022) Exploring the diversity of *Eupolyphaga* Chopard, 1929 (Blattodea, Corydioidea): Species delimitation based on morphology and molecular analysis. ZooKeys 1120: 67–94. 10.3897/zookeys.1120.87483 36760327 PMC9848677

[B3] Han W Che YL Zhang PJ Wang ZQ (2024a) New species of *Eupolyphaga* Chopard, 1929 and *Pseudoeupolyphaga* Qiu & Che, 2024 (Blattodea, Corydioidea, Corydiinae), with notes on their female genitalia. ZooKeys 1211: 151–191. 10.3897/zookeys.1211.128805 39268010 PMC11391126

[B4] Han W Qiu L Zhang JW Wang ZQ Che YL (2024b) Phylogenetic reconstruction of Corydioidea (Dictyoptera: Blattodea) provides new insights on the placement of Latindiinae and supports the proposal of the new subfamily Ctenoneurinae. Systematic Entomology 19(1): 156–172. 10.1111/syen.12610

[B5] Kimura M (1980) A simple method for estimating evolutionary rates of base substitutions through comparative studies of nucleotide sequences. Journal of Molecular Evolution 16(2): 111–120. 10.1007/BF01731581 7463489

[B6] Klass KD (1997) The external male genitalia and the phylogeny of Blattaria and Mantodea. Bonner Zoologische Monographien 42: 1–341.

[B7] Kumar S Stecher G Tamura K (2016) MEGA7: Molecular evolutionary genetics analysis version 7.0 for bigger datasets. Molecular Biology and Evolution 33(7): 1870–1874. 10.1093/molbev/msw054 27004904 PMC8210823

[B8] Lanfear R Frandsen PB Wright AM Senfeld T Calcott B (2017) PartitionFinder 2: New methods for selecting partitioned models of evolution for molecular and morphological phylogenetic analyses. Molecular Biology and Evolution 34: 772–773. 10.1093/molbev/msw260 28013191

[B9] Qiu L Che YL Wang ZQ (2018) A taxonomic study of *Eupolyphaga* Chopard, 1929 (Blattodea: Corydiidae: Corydiinae). Zootaxa 4506(1): 1–68. 10.11646/zootaxa.4506.1.1 30485992

[B10] Roth LM (2003) Systematics and phylogeny of cockroaches (Dictyoptera: Blattaria). Oriental Insects 37(1): 1–186. 10.1080/00305316.2003.10417344

